# A Protein Binding Specifically to the IgG2b Switch Region

**DOI:** 10.1155/1997/72932

**Published:** 1997

**Authors:** Heiner Völk, Matthias Wabl

**Affiliations:** 1 Department of Microbiology and Immunology University of California San Francisco 94143-0670 USA; 2 MRC Cell Mutation Unit University of Sussex BNI9RR United Kingdom

**Keywords:** Immunoglobulin, class switch, nuclear extracts, affinity chromatography, gel shifts, switch recombinase

## Abstract

The Abelson-virus-transformed mouse pre-B-cell line 18-81 switches almost exclusively from μ to γ2b. From nuclear extracts of this cell line, we have isolated a factor that specifically binds to S_γ2b_. After an eight-step purification scheme, in which different types of DNA-affinity chromatography were used as key elements, we obtained a preparation with two narrowly spaced bands at approximately 69 kD on a silver-stained SDS gel. Binding specificity of main-peak fractions of affinity-purified proteins was analyzed by gel shift assays in which S_γ2b_, but not S_μ_, competes. The results are consistent with this factor being part of the switch recombinase.


*Developmental Immunology*, 1997, Vol. 5, pp. 105–114
Reprints available directly from the publisher
Photocopying permitted by license only

© 1997 OPA (Overseas Publishers Association)
Amsterdam B.V. Published under license in The Netherlands
under the Harwood Academic Publishers imprint
part of The Gordon and Breach Publishing Group
Printed in Malaysia

### A Protein Binding Specifically to the IgG2b Switch Region

HEINER VÖLK* and MATTHIAS WABL

*Department of Microbiology and Immunology, University of California, San Francisco, 94143-0670*

*(Received 20 August 1996; Revised 2 October 1996)*

The Abelson-virus-transformed mouse pre-B-cell line 18–81 switches almost exclusively from $\mu$ to $\gamma$2b. From nuclear extracts of this cell line, we have isolated a factor that specifically binds to $S_{\gamma 2b}$. After an eight-step purification scheme, in which different types of DNA-affinity chromatography were used as key elements, we obtained a preparation with two narrowly spaced bands at approximately 69 kD on a silver-stained SDS gel. Binding specificity of main-peak fractions of affinity-purified proteins was analyzed by gel shift assays, in which $S_{\gamma 2b}$, but not $S_\mu$, competes. The results are consistent with this factor being part of the switch recombinase.

*Keywords:* Immunoglobulin, class switch, nuclear extracts, affinity chromatography, gel shifts, switch recombinase

### INTRODUCTION

The earliest antibodies produced during the course of an immune response are of the class IgM. As the response proceeds, antibodies with the same specificity of other classes are produced and IgM production declines. This heavy- (H-) chain class switch is not a population phenomenon; a committed B cell that produces an IgM antibody can switch to the production of another class of immunoglobulin (Wabl et al., 1978). The molecular basis of the H-chain class switch is the deletion of a stretch of DNA from 5′ of $C_\mu$ to 5′ of the $C_H$ gene segment to be expressed (Honjo and Kataoka, 1978; Jäck et al., 1988; Iwasato et al., 1990; von Schwedler et al., 1990). The deletion results from a genetic exchange, with the deleted material being excised as a circular DNA molecule, the so-called switch circle (Jäck et al., 1988; Iwasato et al., 1990; von Schwedler et al., 1990; Harriman et al., 1993). Most recombination break points lie within the so-called switch (S) regions. These are repetitive DNA sequences 5′ of all $C_H$ gene segments except $C_\delta$ (Shimizu and Honjo, 1984). Their overall length (1–10 kb), as well as the length of their repeat units, is variable. However, all switch regions share some homology. By using $S_\mu$ as reference sequence, the degree of homology decreases in the following order: $S_\mu > S_\varepsilon > S_\alpha > S_{\gamma 3} > S_{\gamma 1} > S_{\gamma 2b} > S_{\gamma 2a}$ (Nikaido et al., 1982; Stanton and Marcu, 1982).

Little is known about the switch recombinase, the putative enzyme-mediating switch recombination. However, such an enzyme complex must perform the three basic functions of binding, cutting, and ligating. In a current model of class switching, the future $C_H$ gene segment is targeted by transcription and/or demethylation (reviewed in Lin et al., 1992; Siebenkotten and Radbruch, 1995; Stavnezer, 1996). This is thought to be mediated by vari-

*Current address: MRC Cell Mutation Unit
University of Sussex
BNI9RR U.K.

ous cytokines produced by T cells (reviewed in Finkelman et al., 1990). Recombination of S regions would then proceed, possibly mediated by the normal recombination and repair machinery. Indeed, evidence has accumulated that so-called germ-line transcripts are necessary for switch recombination (Lennon and Perry, 1985; Stavnezer-Nordgren and Sirlin, 1986; reviewed in Lin et al., 1992; Lorenz et al., 1995). But other loci are being transcribed as well, so switch recombination needs to be restricted to S regions; that is, the transcripts themselves may take part in the reaction (Lorenz et al., 1995), or their translation product (Bachl et al., 1996).

Recombination of an isolated (Borggrefe et al., 1996) or transfected (Daniels and Lieber, 1995) switch substrate now can be assayed *in vitro*, and this has revealed a dependence of the switch recombination on transcription (Daniels and Lieber, 1995). There has been some progress in defining consensus recognition sites by analysis of DNA sequences around recombination break points (Wuerffel et al., 1992; Chou and Morrison, 1993; Kenter et al., 1993), but a more extensive analysis has failed to find such a sequence (Dunnick et al., 1993). In the last few years, there have been reports of several factors that bind to various switch regions, and sometimes to other loci as well, in humans and mice (Wuerffel et al., 1990; Schultz et al., 1991; Xu et al., 1992; Fukita et al., 1993; Mizuta et al., 1993; Kenter et al., 1993). To our knowledge, no function in switch recombination has been shown for these proteins. To date, only the genes encoding a factor that binds single-stranded DNA related to $S_\mu$ have been cloned from mouse and humans (Fukita et al., 1993; Mizuta et al., 1993). Two other polypeptides were shown to be transcription factors with targets located also outside of switch regions (Waters et al., 1989; Barberis et al., 1990; Schultz et al., 1991; Williams and Maizels, 1991; Liao et al., 1992; Williams et al., 1993; Brys and Maizels, 1994; Liao et al., 1994; Neurath et al., 1994).

We have assumed that specificity during switch recombination is not entirely determined by cytokine specificity, that is, that specificity is also conferred by factors that bind to S regions. Nontransformed pre-B cells do not seem to switch at all. Nevertheless, all mouse Abelson-virus transformed pre-B cells switch almost exclusively from μ to γ2b (Burrows et al., 1981, 1983; Alt et al., 1982; Akira et al., 1983). If our assumption is correct, nuclear extracts from such transformed cells ought to contain factors that predominantly recognize Sγ2b. Here we describe the isolation of one such factor.

### RESULTS AND DISCUSSION

Proteins binding to $S_{\gamma 2b}$ were purified from nuclear extracts of the Abelson-virus transformed mouse cell line 18–81. Gel shift assays and silver-stained SDS gels were used to screen the fractions at each stage of purification.

### Purification and Gel Shift Assays

Nuclear extracts (200 mg) were loaded onto a 106-ml DEAE fast-flow Sepharose column, eluted with a 400-ml linear gradient from 100 to 200 mM NaCl, followed by an additional 200 mM wash. On the basis of a gel shift analysis (Fig. 1), fractions 36–96 eluting at 111–195 mM and containing 10.8 mg protein were

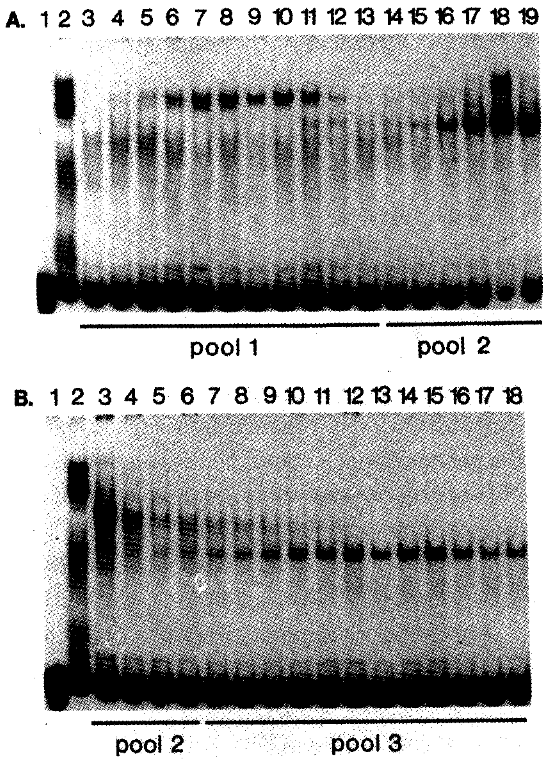

FIGURE 1 Autoradiograms of gel shift assays with eluates from the 106-ml DEAE column. Every fourth fraction was probed with a 57-bp $S_{\gamma 2b}$ fragment. In each gel, lane 1 had no protein; lane 2 had 13.4 μg crude nuclear extract. (A) Lanes 3–19: Fractions 54–118. (B) Lanes 3–18: Fractions 122–182. Fractions 36–96 (pool 1) were processed further.

pooled, diluted to 100 mM NaCl, and passed over a 50-ml BioRex 70 column. Bound material was released with a linear 300-ml 100–1000 mM linear gradient followed by a 2 M NaCl wash. Fractions 16–40 eluting at 100–310 mM NaCl and containing 1.1-mg protein (NaCl) were pooled, concentrated on a 1-ml DEAE column, and loaded in 50 mM NaCl onto a Streptavidin-agarose-$S_{\gamma 2b}$-DNA column (SAS). After elution with 250 mM NaCl and adjustment of pooled active fractions to the proper loading conditions, the material was passed a second time over the SAS column under the same conditions. Pooled active fractions were adjusted to 50 mM NaCl and applied to a third affinity column, which was eluted in steps of 100, 150, 200, and 250 mM NaCl. The 100- and 150-mM eluates contained different gel shift activities, which were well separated from each other (not shown); only the former was studied further. Silver-stained SDS gels of the 100 mM NaCl fractions are shown in Fig. 2.

Active samples from 100 mM NaCl eluates were pooled, diluted to 50 mM, and applied to a fourth SAS column, which was successively eluted with buffer containing 65, 75, and 100 mM NaCl. Silver-stained SDS gels are shown in Fig. 3. DNA-binding activity was detectable in the first two steps only after concentration. In the 100 mM NaCl eluates, the activity peak was maximal at fractions 76–78. The activity decreased rapidly toward the inactive fraction 70 and much more slowly toward the inactive fraction 96. There were few proteins left in the shift-positive fractions, which we will call "SAS-purified" fractions hereafter. The major band is about 69 kD.

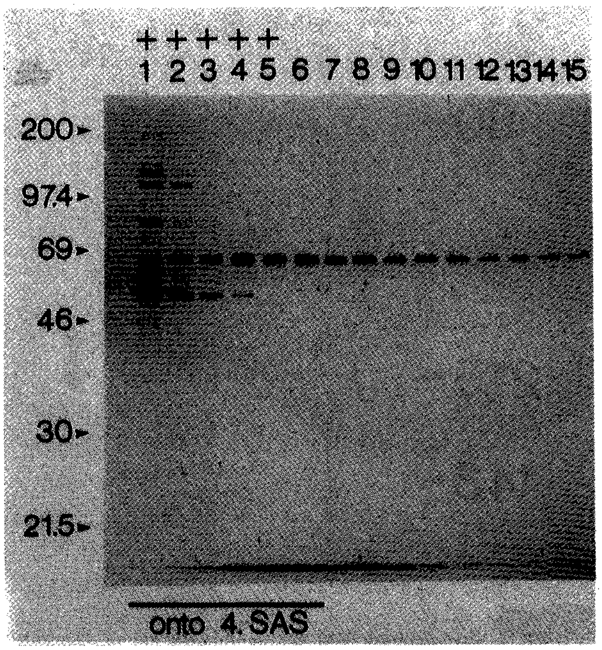

FIGURE 2 Silver-stained gels of eluates from the third affinity column. Aliquots of 30 µl were analyzed under reducing conditions. Molecular weight standards (kD) are indicated on the left. Lanes 1–15: Fractions 14–42 (100 mM NaCl). + : Shift activity.

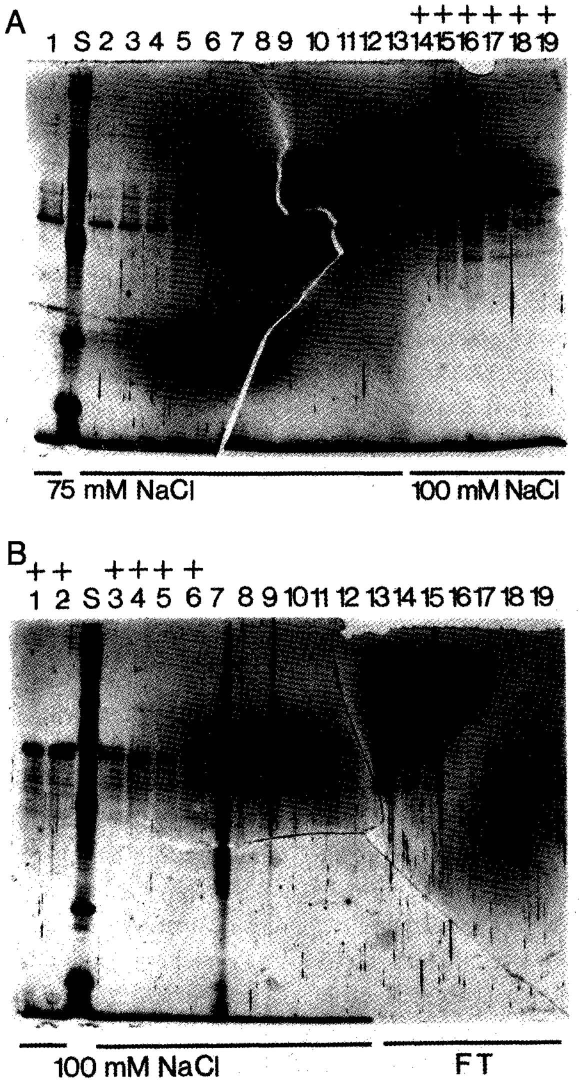

FIGURE 3 Silver-stained gels of SAS-purified fractions. Aliquots of 30 µl were analyzed under reducing conditions. Even fractions were tested. (A) Lanes 1–13: Fractions 46–70 (75 mM NaCl). Lanes 14–19: Fractions 72–82 (100 mM NaCl). (B) Lanes 1–12: Fractions 84–106 (100 mM NaCl). Lanes 13–19: Flowthrough fractions 8, 12–32. + : Shift activity.

### Molecular-Weight Determination after Dynabead Separation

To confirm that the 69-kD protein is indeed the major binding component in these fractions, we ran an SDS gel with Dynabead-purified material. Dynabeads are paramagnetic particles embedded in polystyrene spheres and can be easily removed from a suspension by means of a strong magnet. Beads to which genomic $S_{\gamma 2b}$ had been coupled were incubated with SAS-purified fractions 83–88 and ca. 2% (v/v) of 73–74. Bound material was eluted with basic buffer containing 300 mM NaCl. In pilot experiments, 300 mM NaCl was shown to inhibit gel shifts with the 245-bp $S_{\gamma 2b}$ probe. Control Dynabeads without DNA were treated identically. Aliquots were analyzed again by reducing SDS-PAGE and silver staining (Fig. 4). Supernatant from beads without $S_{\gamma 2b}$ DNA had two narrow-spaced weak bands of ca. 69 kD (Fig. 4, lane 2), which were not visible in the supernatant incubated with DNA-coated beads (Fig. 4, lane 1). The eluate of the $S_{\gamma 2b}$ beads showed a strong narrowly spaced doublet at 69 kD (Fig. 4, lane 3). Although difficult to see on a print, where they appear as a single band, it was easily visible on the original gel that the upper band was slightly weaker. In contrast, the control using beads without $S_{\gamma 2b}$ DNA contained no stainable protein (Fig. 4, lane 4).

### Specificity for $S_{\gamma 2b}$

For competition experiments, fractions 75–78 from the fourth SAS were incubated with end-labeled 245-bp $S_{\gamma 2b}$ fragment and various amounts of various cold DNA fragments in 100 mM NaCl (Fig. 5): There was strong competition with 2.75 ng cold 245-bp $S_{\gamma 2b}$ fragment, and 11 ng virtually abolished any binding of protein to a labeled probe (Fig. 5A). There was no competition with 44 ng of pdIdC, a synthetic product that is supposed to soak up unspecific binders to the DNA backbone (Fig. 5B); nor with 44 ng PhiX 174 phage RF DNA digested with Hae III (Fig. 5C). More importantly, 24 ng of a 1.3-kb genomic $S_{\mu}$ switch-region fragment did not compete (Fig. 5D). Although a 196-bp pUC piece competed to some extent (Fig. 5A) to reach the same degree of competition as a cold Sγ2b fragment, about four times as much DNA was required (11

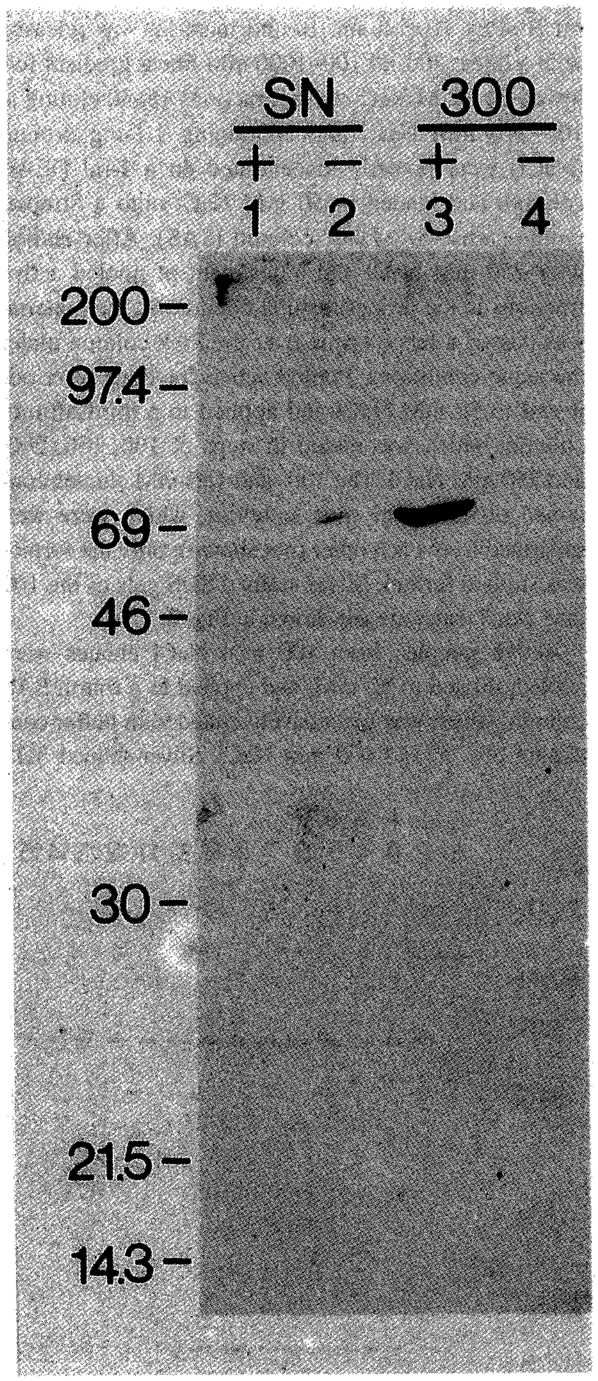

FIGURE 4 Silver-stained SDS polyacrylamide gel of Dynabead purified material: Aliquots of 100 μl were run under reducing conditions; numbers on the left indicate positions of molecular-weight standards. Lanes 1 and 3: Dynabeads coupled to 3.7-kb genomic $S_{\gamma 2b}$ DNA ( + ). Lanes 2 and 4: Dynabeads without DNA ( − ). SN: Supernatant (1.4 ml); 300: 300 mM NaCl eluates (120 μl).

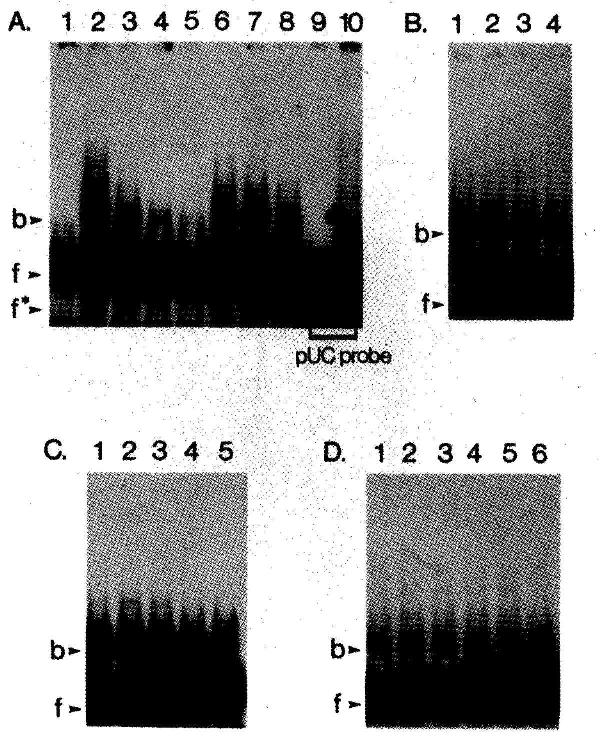

FIGURE 5 Autoradiograms of competition gel shift assays with SAS-purified protein. Unless stated otherwise, the labeled probe was 245-bp $S_{\gamma 2b}$ DNA. (A) Lane 1: No protein. Lane 2: Protein but no competitor. Lanes 3–5: 2.75, 5.5, 11 ng cold 245-bp $S_{\gamma 2b}$ DNA. Lanes 6–8: 2.75, 5.5, 11 ng cold 196-bp pUC DNA. Lane 9: 196-bp labeled pUC probe without protein. Lane 10: 196-bp labeled pUC probe with protein. (B) Lanes 1–4: 5.5, 11, 22, 44 ng cold pdIdC. (C) Lanes 1–5: 2.75, 5.5, 11, 22, 44 ng cold double-stranded PhiX 174 fragments. (D) Lanes 1–6: 4, 8, 12, 16, 20, 24 ng cold 1.3-kb $S_{\mu}$. B: Double-stranded 245-bp $S_{\gamma 2b}$ probe bound to protein. f: Free double-stranded 245-bp $S_{\gamma 2b}$ probe. f*: Free double-standed 196-bp pUC probe.

vs. 2.75 ng). Competition by the pUC piece was somewhat puzzling, especially because when the same pUC fragment was used as a labeled probe, almost no binding activity was detectable (Fig. 5A, lanes 9 and 10). There is some, albeit quite limited, homology between $S_{\gamma 2b}$ and pUC sequences (Table 1).

In another experiment with the 245-bp $S_{\gamma 2b}$ probe in 80 mM NaCl, up to 18 ng of five 57-bp DNAs in combination with 12-ng pdIdC were used. $S_{\gamma 2b}$, $S_{\alpha}$, $S_{\mu}$, and NFκB consensus sequences did not compete, nor did part of a $C_{\mu}$ exon (not shown).

We also tested binding of the 69-kD complex to single-stranded DNA. The 245-bp $S_{\gamma 2b}$ probe was heat-denatured and incubated with protein from fractions 76–77 of the fourth SAS. Little, if any, protein/single-stranded DNA complex was detected (Fig. 6, Lane 2).

To exclude a possible formation of bands by binding of protein to residual nucleotides from labeling reactions, radioactive dCTP or ddATP was incubated with fractionated material in the presence or absence of unlabeled DNA. None of the samples showed any trace of complex formation (not shown).

SAS-purified fractions 74, 79, and 80 were used for DNase I footprinting. Only one very small site was protected at positions 237–238 (CC) of the bottom strand (Fig. 7, lanes 1 and 2). Because the flanking positions were insensitive to DNase I cleavage, even without added fraction material (Fig. 7, lane 1), the actual contact site most likely stretches from positions 236 to 240 (TCCCA). Two other sites with the TCCCA motif were found within the 245-bp $S_{\gamma 2b}$ probe (Table 1), but no protection could be demonstrated under the conditions tested. However, their neighboring sequences showed some diversity, and some of these surrounding bases

TABLE I Comparison of Footprint Sequence (TCCCA) and Flanking Sequences with Similar Motifs and Their Flanking Sequences without DNase I Protection

| DNA | Position[a] | Footprint[b] | Sequence of the the top strand |
|---|---|---|---|
| 196-bp pUC | B 6–30 | ND | GCGCA ACTGT TGGGA AGGGC GATCG |
| 196-bp pUC | T 52–76 | ND | ACGTC GTGAC TGGGA AAACC CTGGC |
| 57-bp $S_{\gamma 2b}$ | B 29–53 | ND | CTGGG GAWGG TRGGA RTRTG ACGCG |
| 245-bp $S_{\gamma 2b}$ | B 22–46 | — | CAGGG ATAGG TGGGA GTATT AGGGA |
| 245-bp $S_{\gamma 2b}$ | B 120–144 | — | CTGGG GCAGG TGGGA GTGTG AGGGA |
| 245-bp $S_{\gamma 2b}$ | B 226–245[c] | + | GGTAG GAATG TGGGA GACCA GATCC |

[a] T/B: Top/Bottom strand has the motif. Positions always refer to the sequence of the top strand.
[b] ND: Not determined.
[c] The last 5 bp of the flanking sequence at the 3′ end are part of the pUC multiple cloning site.

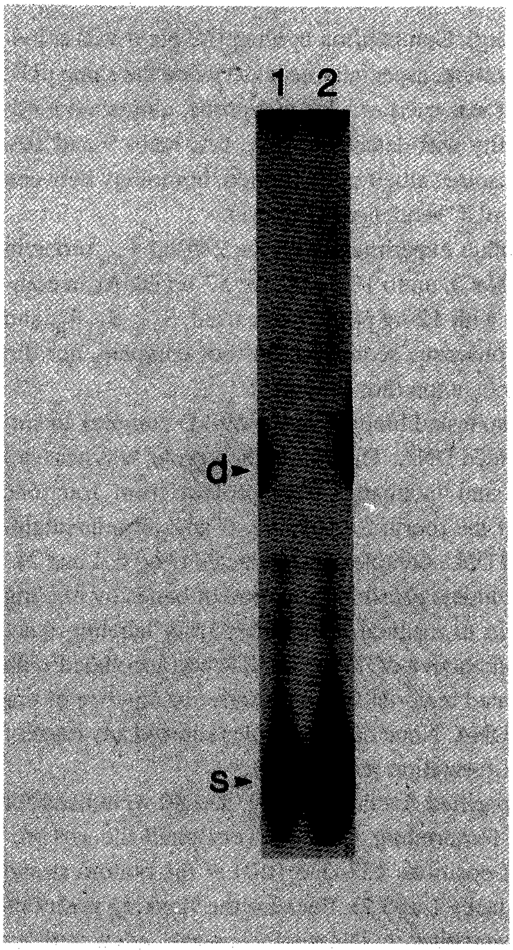

FIGURE 6 Autoradiogram of gel shift analysis with single-stranded DNA. The 245-bp $S_{\gamma 2b}$ probe was heat-denatured and subsequently incubated in 100 mM NaCl buffer without (lane 1) or with (lane 2) material from SAS-purified fractions 76–77. s: Free single-standed DNA probe. d: Position where the free double-stranded probe would run.

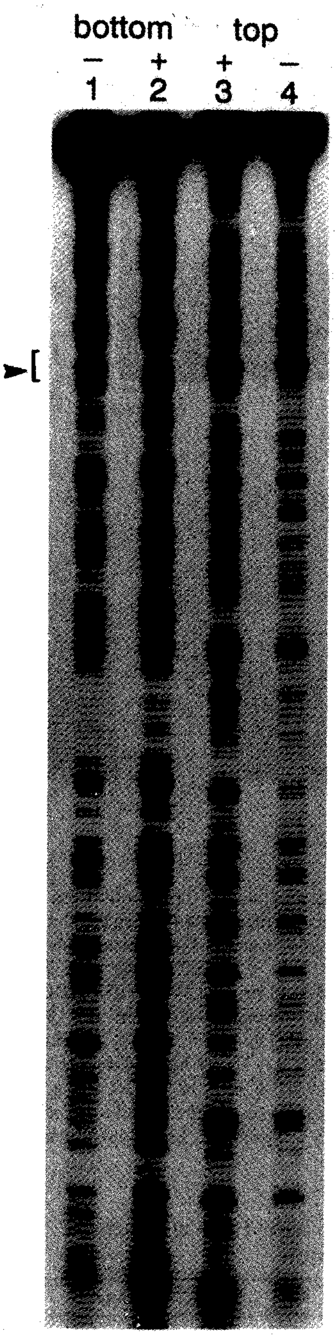

FIGURE 7 Autoradiogram of a DNase I footprinting experiment with subsequent denaturing polyacrylamide gel electrophoresis: Lanes 1 and 2: Bottom strand. Lanes 3 and 4: Top strand. Positions refer to the complementary sequence on the top strand. Samples were incubated with (lanes 2 and 3) or without (lanes 1 and 4) SAS-purified protein. Subsequent denaturing electrophoresis was done for 4 hr. The arrowhead indicates protection at positions 237–238 (CC) of the bottom strand.

could have an effect on directing DNA-binding proteins to the one but not the other site. Another reason of why there was no large footprint or no other visible footprints could have been the decreased binding of purified proteins in the presence of divalent cations. But calcium and magnesium ions need to be added to the binding mix together with DNase I in order to promote the nicking reaction. It may be that very high concentrations of purified protein will be necessary to reach a reasonable balance.

Using the data on competition with the pUC fragment, and assuming the 5-bp stretch as the complete and only site in the probe, one can estimate the competitiveness ratio of specific to nonspecific DNA in the gel shift assays to be 110. That is, the specific 5-bp motif is 110 times as good a competitor as nonspecific DNA.

### Does Our 69-kD Factor Correspond to a Published One?

Kenter et al. (1993) described sites for two factors called SNAP and SNIP/NFκB, which bind to sites A and B, respectively. These sites can be found in the switch regions $S_{\gamma 3}$, $S_{\gamma 1}$, and $S_{\gamma 2b}$. The 245-bp $S_{\gamma 2b}$ probe used in our experiments has three potential 15-bp spanning A sites at positions 8–22, 57–71, and 204–218. There is only one mismatch, which is located within the first site. SNAP, of unknown molecular weight, is very pH-sensitive and does not seem to bind to DNA above 150 mM NaCl. This seems to distinguish it from the 69-kD factor, although pH dependence and salt concentrations for elutions are difficult to compare. Attempts to identify SNAP activity in cell line 18–81 were not successful (Amy Kenter, personal communication). For the 69-kD factor reported here, binding at pH 7.4 was only little less than binding at pH 7.9. In addition, our protein-DNA complexes were shown to be more stable in salt; they disappear only between 250 and 300 mM NaCl. Finally, the DNase I protected positions were 236–240, a region well separated from the nearest possible A site at positions 204–218. Thus, our 69-kD protein does not seem to be SNAP.

The B site, which is recognized by NFκB, spans 11 bp. Based on competition gel shifts and a supershift induced by polyclonal anti-p50 serum, it was proposed that NFκB is involved in binding to various switch regions. NFκB consists of a p50 and p65 subunit with molecular weights of 50 and 65 kD, respectively. Heterodimers consisting of p50 and p65, as well as p50 homodimers, can bind to DNA (Urban et al., 1991). The $S_{\gamma 2b}$ probe used in our experiments has two potential B sites at positions 42–52 and 140–150 (one mismatch each). However, there are several reasons why we think the purified shift activities from our work are different from NFκB. In competition gel shift assays, a 57-bp DNA containing three complete NFκB sites should have been a very strong competitor, but it did not compete. The 196-bp pUC fragment contains one complete NFκB site, but used as a probe, it hardly bound anything. Further, our footprinting experiments show a protected site at position 236–240, which is far away from the two potential B sites at positions 42–52 and 140–150. Thus, our 69-kD protein does not seem to be SNIP/NFκB. Nor is it likely that it is the LR-1 factor, which has been described to be a 106-kD protein (Williams et al., 1993).

We conclude that the 69-kD factor that specifically binds to $S_{\gamma 2b}$ is likely to be a novel protein.

### MATERIALS AND METHODS

### Nuclear Extracts

Cells of the Abelson-virus-transformed mouse pre-B-cell line 18–81 were grown to 0.5 to 2 $\times$ $10^6$/ml. Nuclear extracts (200 mg) were prepared from 26 l of cells according to Ausubel et al. (1987) with the following modifications: All buffers contained 1 mM PMSF, 1 μg/ml Leupeptin, Pepstatin, and Aprotinin. Hypotonic buffer for swelling as well as all subsequently used buffers were supplemented with 0.1% NP-40. The final NaCl concentration during the 45-min extraction was 405 mM. Before freezing, samples were adjusted to 100 mM NaCl and 20% glycerol by dilution rather than by dialysis.

### Column Chromatography

All steps were performed at 4°C. The basic buffer was 20 mM HEPES, pH 7.9, 20% glycerol, 1 mM DTT, 0.1 mM EDTA, 1 mM PMSF, as well as 1 μg/ml E-64, Leupeptin, Pepstatin, and Aprotinin. Flowthrough, washes, and eluted fractions were screened for DNA-binding activity by gel shift assays. Determination of protein concentrations was done with Bradford assays.

To prepare the SAS affinity column, 5.1 ml Streptavidin agarose was washed with 2 M NaCl, TE, 0.1% NP-40 followed by PBS. Biotinylated (double-stranded) 57-bp $S_{\gamma 2b}$ DNA (300 μg) in 3 ml fill-in mix with 0.1% NP-40 was passed over the column four times before washing with PBS, 0.1% NP-40, 1 mM EDTA followed by 2 M NaCl, TE, 0.1% NP-40, then 100 mM

NaCl, TE at pH 8.0. An equilibration step was performed with basic buffer supplemented with 50 mM NaCl, NP-40, BSA, DTT, and Bestatin. Before loading, samples were adjusted to 50 mM NaCl, 0.1% NP-40, 0.1 mg/ml BSA, and 40 μg/ml Bestatin, unless otherwise specified. Washes and elution were performed without BSA. Bound material was eluted with step gradients.

### Purification with Dynabeads Coupled to Genomic $S_{\gamma 2b}$ DNA

The 3.7-kb insert of the plasmid p245 was released by digest with the restriction enzymes Eco RI and Hin dIII. Biotinylation of the Eco RI end was accomplished by partial Sequenase fill-in with dATP and Biotin-16-ddUTP. DNA (72 μg) was coupled to 1 ml of Dynabeads suspension (6–7 $\times 10^8$/ml) in TE, 1 M NaCl for 2 hr. Before and after coupling, beads were washed extensively with the same buffer used for immobilization of the DNA. Ca. 1.4 ml SAS-purified protein, mostly fractions 83–88 and only about 2% (v/v) 73–74, were incubated with the beads for 7 hr on ice with occasional agitation. Elution was performed for 20 min on ice with 120 μl basic buffer containing 300 mM NaCl.

### Gel Shift Assays

The gel shift assay was modified from Strauss and Varshavsky (1984). Protein was incubated with 10,000 cpm of 3′ end-labeled probe and various amounts of pdIdC for 20 min at RT. The final volume was 20 μl in 20 mM HEPES pH 7.9, 10–14% glycerol, 1 mM DTT, and 100 mM NaCl. Samples were electrophoresed for 10 min at 200 V at RT through a 4% TAE polyacrylamide gel and then at 180 V for 1.5–2.5 hr at 4°C.

### DNAs and DNA Sequencing

#### *Plasmids*

pUC/Sμ (A), a gift from Richard Scheuermann (Dallas), is a derivative of pUC 19 with the 3.5-kb Xba I fragment 5′ to the $C_{\mu}$ exons (Stanton and Marcu, 1982). The insert with the switch μ region was modified with linkers and cloned into the Cla I site. $pS_{\gamma 2b}$ is a pUC 19 with the insert of the plasmid pSL1 (Lang et al., 1982) with a ca. 400-bp deletion at the 3′ end of the insert. p245 is a pUC 19 containing the 245-bp Bgl II subfragment from the $pS_{\gamma 2b}$ insert cloned into the Bam HI site.

| | |
|---|---|
| **17-mer Primer mcs1** | CGAGCTCGGTACCCGGG |
| **17-mer Primer mcs2** | TGCATGCCTGCAGGTCG |
| **57-mer $S_{\gamma 2b}$** (Nikaido et al., 1982) | GGGACCAGWCCTAGCAGCTPTGGGGGAGCTGGGGAWGGTPGGAPTPTGACGCGATTA |
| **57-mer $S_{\alpha}$** (Nikaido et al., 1982) | ATGAGCTGGGATGAGCTGAGCTAGGCTGGAATAGGCTGGGCTGGGCTGGCGCGATTA |
| **57-mer $S_{\mu}$** (Nikaido et al. 1982) | GAGCTGAGCTGGGGTGAGCTGAGCTGAGCTGGGGTGAGCTGAGCTGAGCCGCGATTA |
| **57-mer NFκB** (Urban et al., 1991) | GAGGGGACTTTCCGAGAGAGGGGACTTTCCGAGAGAGGGGACTTTCCGACGCGATTA |
| **57-mer $C_{\mu}$** (Schreier et al., 1986) | GGGACTTCCTGCCCAGCACCATTTCTTCACCTGGAACTACCAGAACAACCGCGATTA |

**8-mer Primer.** (Bio)-TAATCGCG synthesized with Biotin-ON phosphoramidite (Clontech).

**pdIdC.** 1.2–1.4 kb average length (Pharmacia).

**PhiX 174 RF** (Sanger et al., 1978).

**DNase I Footprinting and AG Chemical Sequencing.** These were done according to Ausubel et al. (1987). Strand scission was done in 10 μl 100 mM NaOH, 1 mM EDTA for 30 min at 90°C. After precipitation, samples were run on a 6%, 7 M urea sequencing gel.

### Acknowledgements

This work was supported by NIH grant RO1 GM37699 and by funds of the Markey Trust, and by fellowships of the Fonds der Chemischen Industrie and the Daimler-Benz Foundation to H.V. Most of the material here is taken from H.V.'s doctoral thesis submitted to the University of Kaiserslautern, Germany.

### References

Akira, S., Sugiyama, H., Yoshida, N., Kikutani, H., Yamamura, Y. and Kishimoto, T. (1983) Isotype switching in murine pre-B cell lines. *Cell*, **34**, 545–556.

Alt, F. W., Rosenberg, N., Casanova, R. J., Thomas, E. and Baltimore, D. (1982) Immuno-globulin heavy-chain expression and class switching in a murine leukemia cell line. *Nature*, **296**, 325–331.

Ausubel, F. M., Brent, R., Kingston, R. E., Moore, D. D., Smith, J. A., Seidman, J. G. and Struhl, K. (1987) In Current Protocols in Molecular Biology (New York: Greene Publishing and John Wiley), Interscience.

Bachl, J., Turck, C. W. and Wabl, M. (1996) Translatable immunoglobulin germ line transcript. *Eur. J. Immunol.*, **26**, 870–874.

Barberis, A., Widenhorn, K., Vitelli, L. and Busslinger, M. (1990) A novel B-cell lineage-specific transcription factor present at early but not late stages of differentiation. *Genes Dev.*, **4**, 849–859.

Brys, A. and Maizels, N. (1994) LR1 regulates c-myc transcription in B-cell lymphomas. *Proc. Natl. Acad. Sci. USA*, **91**, 4915–4919.

Burrows, P. D., Beck, G. B. and Wabl, M. R. (1981) Expression of μ and γ immunoglobulin heavy chains in different cells of a cloned mouse lymphoid line. *Proc. Natl. Acad. Sci. USA*, **78**, 564–568.

Burrows, P. D., Beck-Engeser, G. B. and Wabl, M. R. (1983) Immunoglobulin heavy-chain class switching in a pre-B cell line is accompanied by DNA rearrangement. *Nature*, **306**, 243–246.

Chou, C. L. and Morrison, S. L. (1993) A common sequence motif near nonhomologous recombination breakpoints involving Ig sequences. *J. Immunol.*, **150**, 5350–5360.

Daniels, G. A. and Lieber, M. R. (1995) Strand specificity in the transcriptional targeting of recombination at immunoglobulin switch sequences. *Proc. Natl. Acad. Sci. USA*, **92**, 5625–5629.

Dunnick, W., Hertz, G. Z., Scappino, L. and Gritzmacher, C. (1993) DNA sequences at immunoglobulin switch region recombination sites. *Nucleic Acids Res.*, **21**, 365–372.

Finkelman, F. D., Holmes, J., Katona, I. M., Urban, J. F., Beckmann, M. P., Park, L. S., Schooley, K. A., Coffman, R. L., Mosmann, T. R. and Paul, W. E. (1990) Lymphokine control of in vivo immunoglobulin isotype selection. *Annu. Rev. Immunol.*, **8**, 303–333.

Fukita, Y., Mizuta, T. R., Shirozu, M., Ozawa, K., Shimizu, A. and Honjo, T. (1993) The human Sμbp-2, a DNA-binding protein specific to the single-stranded guanine-rich sequence related to the immunoglobulin μ chain switch region. *J. Biol. Chem.*, **268**, 17463–17470.

Harriman, W., Volk, H., Defranoux, N. and Wabl, M. (1993) Immunoglobulin class switch recombination. *Annu. Rev. Immunol.*, **11**, 361–384.

Honjo, T. and Kataoka, T. (1978) Organization of immunoglobulin heavy chain genes and allelic deletion model. *Proc. Natl. Acad. Sci. USA*, **75**, 2140–2144.

Iwasato, T., Shimizu, A., Honjo, T. and Yamagishi, H. (1990) Circular DNA is excised by immunoglobulin class switch recombination. *Cell*, **62**, 143–149.

Jäck, H. M., McDowell, M., Steinberg, C. M. and Wabl, M. (1988) Looping out and deletion mechanism for the immunoglobulin heavy-chain class switch. *Proc. Natl. Acad. Sci. USA*, **85**, 1581–1585.

Kenter, A. L., Wuerffel, R., Sen, R., Jamieson, C. E. and Merkulov, G. V. (1993) Switch recombination breakpoints occur at nonrandom positions in the $S_\gamma$ tandem repeat. *J. Immunol.*, **151**, 4718–4731.

Lang, R. B., Stanton, L. W. and Marcu, K. B. (1982) On immunoglobulin heavy chain class switching: Two γ2b genes are rearranged via switch sequences in MPC-11 cells but only one is expressed. *Nucleic Acids Res.*, **10**, 611–630.

Lennon, G. G. and Perry, R. P. (1985) $C_\mu$-containing transcripts initiate heterogeneously within the IgH enhancer region and contain a novel 5' non-translatable exon. *Nature*, **318**, 475–478.

Lin, Y. C., Shockett, P. and Stavnezer, J. (1992) Regulation of transcription of the germline immunoglobulin alpha constant region gene. *Curr. Top. Microbiol. Immunol.*, **182**, 157–165.

Liao, F., Birshtein, B., Busslinger, M. and Rothman, P. (1994) The transcription factor BSAP (NF-HB) is essential for immunoglobulin germ-line transcription. *J. Immunol.*, **152**, 2904–2917.

Liao, F., Giannini, S. L. and Birshtein, B. K. (1992) A nuclear DNA-binding protein expressed during early stages of B cell differentiation interacts with diverse segments within and 3' of the IgH chain gene cluster. *J. Immunol.*, **148**, 2909–2917.

Lorenz, M., Jung, S. and Radbruch, A. (1995) Switch transcripts in immunoglobulin class switching. *Science* **267**, 1825–1828.

Mizuta, T. R., Fukita, Y., Miyoshi, T., Shimizu, A. and Honjo, T. (1993) Isolation of cDNA encoding a binding protein specific to 5'-phosphorylated single-stranded DNA with G-rich sequences. *Nucleic Acids Res.*, **21**, 1761–1766.

Neurath M. F., Strober, W. and Wakatsuki, Y. (1994) The murine Ig 3' α enhancer is a target site with repressor function for the B cell lineage-specific transcription factor BSAP (NF-HB, Sα BP). *J. Immunol.*, **153**, 730–742.

Nikaido, T., Yamawaki-Kataoka, Y. and Honjo, T. (1982) Nucleotide sequences of switch regions of immunoglobulin Cε and Cγ genes and their comparison. *J. Biol. Chem.*, **257**, 7322–7329.

Sanger, F., Coulson, A. R., Friedmann, T., Air, G. M., Barrell, B. G., Brown, N. L., Fiddes, J. C., Hutchison, C. A., Slocombe, P. M. and Smith, M. (1978) The nucleotide sequence of bacteriophage phi X 174. *J. Mol. Biol.*, **125**, 225–246.

Schreier, P. H., Quester, S. and Bothwell, A. (1986) Allotypic differences in murine μ genes. *Nucliec Acids Res.*, **14**, 2381–2389.

Schultz, C. L., Elenich, L. A. and Dunnick, W. A. (1991) Nuclear protein binding to octamer motifs in the immunoglobulin γ1 switch region. *Int. Immunol.*, **3**, 109–116.

Shimizu, A. and Honjo, T. (1984). Immunoglobulin class switching. *Cell* **36**, 801–803.

Siebenkotten, G. and Radbruch, A. (1995). Towards a molecular understanding of immunoglobulin class switching. *The Immunologist*, **3**, 141–145.

Stanton, L. W. and Marcu, K. B. (1982) Nucleotide sequence and properties of the murine γ3 immunoglobulin heavy chain switch region: Implications for successive $C_\gamma$ gene switching. *Nucleic Acids Res.*, **10**, 5993–6006.

Stavnezer, J. (1996). Immunoglobulin class switching. *Curr. Opin. Immunol.*, **8**, 199–205.

Stavnezer-Nordgren, J. and Sirlin, S. (1986) Specificity of immunoglobulin heavy chain switch correlates with activity of germline heavy chain genes prior to switching. *EMBO J.*, **5**, 95–102.

Strauss, F. and Varshavsky, A. (1984). A protein binds to a satellite DNA repeat at three specific sites that would be brought into mutual proximity by DNA folding in the nucleosome. *Cell*, **37**, 889–901.

Urban, M. B., Schreck, R. and Baeuerle, P. A. (1991) NF-κB contacts DNA by a heterodimer of the p50 and p65 subunit. *EMBO J.*, **10**, 1817–1825.

von Schwedler, U., Jäck, H. M. and Wabl, M. (1990) Circular DNA is a product of the immunoglobulin class switch. *Nature*, **345**, 452–454.

Wabl, M. R., Forni, L. and Loor, F. (1978). Switch in immunoglobulin class production observed in single clones of committed lymphocytes. *Science*, **199**, 1078–1080.

Waters, S. H., Saikh, K. U. and Stavnezer, J. (1989) A B-cell-specific nuclear protein that binds to DNA sites 5' to immunoglobulin $S_\alpha$ tandem repeats is regulated during differentiation. *Mol. Cell. Biol.*, **9**, 5594–5601.

Williams, M. and Maizels, N. (1991). LR1, a lipopolysaccharide-responsive factor with binding sites in the immunoglobulin switch regions and heavy-chain enhancer. *Genes Dev.*, **5**, 2353–2361.

Williams, M., Hanakahi, L. A. and Maizels, N. (1993). Purification and properties of LR1, an inducible DNA binding protein from mammalian B lymphocytes. *J. Biol. Chem.* **268**, 13731–13737.

Wuerffel, R. A., Asher, T. N. and Kenter, A. L. (1990). Detection of an immunoglobulin switch region-specific DNA-binding protein in mitogen-stimulated mouse splenic B cells. *Mol. Cell. Biol.* **10**, 1714–1718.

Wuerffel, R., Jamieson, C. E., Morgan, L., Merkulov, G. V., Sen, R. and Kenter, A. L. (1992) Switch recombination breakpoints are strictly correlated with DNA recognition motifs for immunoglobulin $S_{\gamma3}$ DNA-binding proteins. *J. Exp. Med.*, **176**, 339–349.

Xu, L., Kim, M. G. and Marcu, K. B. (1992) Properties of B cell stage specific and ubiquitous nuclear factors binding to immunoglobulin heavy chain gene switch region. *Int. Immunol.* **4**, 875–887.

